# Pyruvate Kinase M2 serves as blockade for nucleosome repositioning and abrogates Chd7 remodeling activity

**DOI:** 10.1371/journal.pone.0211515

**Published:** 2019-02-08

**Authors:** Kirtika Verma, Ashok Patel

**Affiliations:** Kusuma School of Biological Sciences, Indian Institute of Technology Delhi, India; Macau University of Science and Technology, MACAO

## Abstract

Pyruvate Kinase M2 (PKM2) mediates metabolic reshuffling and is ubiquitously upregulated in several cancer types. The non-metabolic function of PKM2 as key nuclear kinase and modulator of gene expression is instrumental in cancer progression and tumorigenesis. Here, we attempt to discern the non-canonical function of PKM2 as an epigenetic modulator and the underlying implication of this activity. Using 5’-FAM labelled reconstituted mononucleosome we have shown that PKM2 interacts with the complex through Histone H3 and possibly obstruct the access to DNA binding factors. Subsequently, the interaction negatively impacts the ATP dependent remodeling activity of Chromodomain Helicase DNA binding protein-7 (Chd7). Chd7 remodeling activity is required to ameliorate DNA damage and is crucial to genome stability. Our study shows that PKM2 blocks the Chd7 mediated sliding of nucleosome. It can be conjectured that stalling Chd7 may lead to impaired DNA damage and increased genomic instability. We propose a mechanism in which PKM2 negatively regulate nucleosome repositioning in chromatin and may exacerbate cancer by altering the nucleosome architecture. This research is imperative to our understanding of how altered cancer metabolism can potentially modulate the gene expression and sustain incessant proliferation by tweaking the chromatin topography.

## Introduction

Metabolism and gene expression are stringently regulated physiological process that are concurrently modulated by a wide array of enzymes and regulatory protein. The concerted control mechanism exerted by the milieu of governing factors lead to dynamic regulation of the cellular machinery and contribute to incessant cell proliferation and tumorigenesis [1]. Metabolic reprogramming characterized by elevated glucose uptake and lactate production is the hallmark of most of the cancer tissue [2]. Pyruvate Kinase enzyme catalyzes the last rate-limiting step of glycolysis converting Phosphoenol Pyruvate to pyruvate with the subsequent production of ATP. Pyruvate Kinase M2 (PKM2) is an oncofetal isoform generated as a result of alternative splicing of the PKM mRNA transcript exhibit low basal activity and thus is a key player in regulating the glycolytic flux contributing to cancer progression [3–6]. It results in the build-up of glycolytic intermediates which are directed towards the biosynthetic processes [7]. PKM2 pyruvate kinase activity is critical for cancer metabolism and can be regulated by posttranslational modifications and patient derived mutations through the tweaking of PKM2 tetramer conformation [8]. Multiple cancer cell lines of different tissue origin exclusively express PKM2 that confers selective growth advantage [9]. This can be attributed to structural dynamicity and oligomeric switch that facilitates nuclear translocation. PKM2 dimer acts as transcriptional co-activator and a key nuclear kinase by directly interacting and phosphorylating several different human proteins as can be inferred from the protein microarray data analysis [10,11]. Phosphorylation of PKM2 Y105 mediated by tyrosine kinases such as FGFR1, Jak2 and BCR-ABL hinder active tetramer conformation, thus resulting in impaired PKM2 enzymatic activity. The dimeric PKM2 undergoes nuclear translocation and initiates a cascade of event [12]. It is known to augment the transcription of cytochrome P450 1A1 (CYP1A1) by serving as complex along with PDC-E2 and (HAT) p300 at the arylhydrocarbon receptor (AhR) [13]. Dimeric PKM2 is reported to upregulate the expression of MEK5 by phosphorylating STAT3 at Y705 [14]. As per study nuclear PKM2 bind Tyr33 phosphorylated β-catenin and enhance its transactivation activity. β-catenin associated PKM2 subsequently phosphorylate histone H3 at T11 and initiate an array of epigenetic events triggering enhanced expression of CCND1 and c-MYC [15,16].

It is intriguing to understand how PKM2 regulate transcription by modifying histones and discern the potential implications of this interaction on the chromatin structure. Reports suggest that a plethora of protein complexes regulate gene expression either by directly modifying histones or tweaking the chromatin structure. [17]. The condensed chromatin restrict the ready access of genomic DNA and inhibit the coordinated chromosomal transactions such as DNA repair, transcription, etc. However this impediment is overcome by via several post translational modifications and remodeling complexes that facilitate ATP driven nucleosome sliding. Translocation necessitates the disruption of all the histone-DNA bonds with the subsequent sliding of DNA from end nucleosome to the dyad or vice-versa and require approximately 12–14 kcal mol^−1^ [18–25].

Chromatin-remodeling enzymes engineer the epigenetic signature of the cell and facilitate a potential triggering of several key cellular pathways by orchestrating nucleosome sliding and altering gene expression. Chd7, a member of chromodomain enzyme family contain the DNA-binding domains namely SANT and SLIDE, and a motor domain that induces hydrolysis of ATP. Studies report that residues flanked by conserved ATPase motor and DNA-binding domain are necessarily not required for the nucleosome-stimulated ATP hydrolysis, nonetheless they facilitate efficient utilization of the energy of ATP hydrolysis for the nucleosome sliding [26–28]. Chd7 is implicated in a number of developmental disorder and is interestingly dysregulated in spectrum of cancer subtypes with aberrant expression, copy number variation, and somatic mutations [29–31]. ATM/ATR checkpoint kinases are implicated in DNA Damage Repair (DDR) with Chd7 as a potential putative substrate. To note, DDR pathways are crucial for genome integrity and any aberration like mutation or epigenetic silencing may lead to cell proliferation and tumorigenesis [32,33].

Here, we show that the PKM2 during in -vitro reconstitution potentially interacts with Histone H3 of the 5’-FAM labelled nucleosome octamer core and forms a stable complex as demonstrated by the native PAGE binding experiment. As has been reported previously PKM2 phosphorylates Histone H3 at T11 [34] our results further validate this interaction at the nucleosome level. Similar results were obtained for a 34-mer multi-unit chromatinized plasmid. To further investigate the nature and possible implication of this interaction, Chd7 mediated nucleosome sliding experiments were performed. As per the finding Chd7 remodeler failed to slide nucleosome off the ends of DNA. We propose that PKM2 abrogates the Chd7 mediated remodeling activity and thereby may exacerbate the genomic integrity and further contribute to cancer initiation and progression.

## Materials and methods

### Protein purification

pET-28a-hPKM2 was a gift from Lewis Cantley & Matthew Vander Heiden (Addgene plasmid # 44242). *Pyruvate Kinase M2****-*** protein was expressed in *Escherichia coli* BL21 (DE3) Rosetta cells using the auto induction method [35] and purified by affinity chromatography using His Trap column (GE Healthcare). Fractions containing PKM2 protein were pooled and dialyzed against 1M GuHCL overnight. Post dialysis the protein was concentrated and further purified by size exclusion chromatography using Superdex 200 16/600 column (GE Healthcare). Human Chd7 hsChd7 baculovirus construct in pFAST Bac vector containing 2997aa was a kind gift from Dr. Robert Kingston, The General Hospital Corp., Massachusetts General Hospital, Boston. Truncated construct ΔChd7(787–2134) was sub cloned in a modified pET28a vector between SalI and Not1 restriction sites which has hexa-histidine tag, Flag tag and a Protease cleavage sequence (LEVLFQ/GP) was incorporated immediately prior to residue 787aa. ΔChd7 expression was carried out in Escherichia coli BL21 (DE3) cells (Invitrogen) containing an additional plasmid Trigger Factor chaperone, overexpression of protein was done by using previously stated auto induction method. Four liters of autoinduction media were inoculated with 40 ml of overnight culture, grown for 2 h at 37°C and further allowed to grow at 18°C for 18 h. Cells were harvested by centrifugation and resuspended in buffer A (50 mM Tris, pH 7.5, 500 mM NaCl, 10% glycerol, 10 mM imidazole) containing 1 mM PMSF and 1 mM DTT. During cell lysis for 30 minutes on ice, lysozyme to a final concentration of 1 mg/ml and DNase 0.01 g/ml was added and disrupted by sonication on ice, and the lysate was clarified by centrifugation at 10,000g for 50 min. The lysate was subjected to affinity chromatography using HisTrap column (GE Healthcare) and protein was eluted with buffer A supplemented with 250 mM imidazole followed by cation exchange chromatography on SPFF column (GE Healthcare) eluted with NaCl gradient. Post this fractions were directly pooled, concentrated and finally purified by using Superdex 200 16/60 column (GE Healthcare). The peak fractions were analyzed on 10% SDS-PAGE, concentrated and stored at -80°C. Protein concentrations were determined by UV absorbance at 280 using extinction coefficient as well as quantification by serial dilution on 10% SDS polyacrylamide gel and comparison against BSA standard. Expression and purification of *Xenopus laevis* histone proteins and subsequent refolding into histone octamers was done as per previously described protocol [36].

#### Nucleosome assembly

The reconstitution of nucleosome using DNA and octamer was followed by web protocol (from the Tsukiyama Laboratory). In brief 5’end fluorescent labelled DNA containing 145-bp Widom 601 positioning sequence [37] was generated by Polymerase chain reaction amplification. Labelled 208 bp fragment generated using 5’-FAM-CAGGATGTATATATCTGACACGTGCCTGG and 3’ATGAACTCGGTGTGAAGAATCATGCTTTCC unlabeled oligos was purified by native PAGE and mixed with Histone octamer in the ratio 1:1.5 for nucleosome reconstitution and dialyzed against a 2M KCl High Salt Buffer. Gradient dialysis was achieved by exchanging High salt buffer with Low Salt containing 200 mM of KCl by calibrating peristaltic pump (Gilson) to a flow rate of 0.1ml/min. The reconstituted nucleosome was purified using continuous–elution electrophoresis Mini Prep Cell Assembly System (Bio-Rad) [38,39]. A 10 kb 34-mer array of 601 repeat sequence with 208-bp plasmid pJ201(34 X 601) and histone octamer were salt gradient dialyzed with an optimal saturation ratio of 1:1.5 for the reconstitution of chromatinized plasmids [37].

### Gel mobility shift assay

The gel electrophoresis mobility shift assay was performed using non-denaturing gel to study the PKM2- Histone (H2A, H2B, H3, and H4) and PKM2- octamer interaction. In brief aliquots of proteins (approximately 10 uM each) were incubated overnight at 4^0^ C. The binding buffer composition was as following: 20mM Hepes-KOH, pH 7.5,50mMKCl, 5mM MgCl_2,_ 1mM DTT, 5% sucrose. The samples were then subjected onto a pre-run (90V, 30 min) 5% native polyacrylamide gel (60:1 acrylamide to bis-acrylamide ratio). Buffer used was chilled β- alanine/ acetic acid pH 6.8 and electrophoresis was performed with terminal reversed at 120 V for 120 min [40–42].

### Fluorescence spectroscopy

PKM2 and Histone H3 interaction was monitored by fluorescence spectroscopy. In brief PKM2 was conjugated with FITC by incubating 1ml of 2mg/ml of protein with approximately 50ul of freshly prepared 1mg/ml of FITC stock solution in DMSO. The reaction was incubated at 4^0^ C in dark for 8hrs. The fluorescein to protein ratio of the product was estimated by measuring the absorbance at 495 nm and 280 nm. The F/P ratio obtained was within the range 0.3 and 1.0. Alteration in microenvironment of FITC labelled PKM2 was studied by titrating increasing concentrations of Histone H3. The fluorescence scan was recorded in a 1 cm path length quartz cuvette using an LS 55 fluorescence spectrometer (PerkinElmer, MA, USA). The excitation and emission slits were kept at 7.5 and 2.5 nm respectively with fluorescence readings as (λ excitation = 495 nm, λ emission = 525nm) with the scan range set as 500nm -570 nm. Data from triplicate reactions were averaged and fitted using Nonlinear Curve Fit (Hill) in Origin 8.0 [43,44].

### Surface plasma resonance spectroscopy

Surface plasmon resonance (SPR) experiment was carried out using Autolab SPR (at Advanced Instrumentation Research Facility, Jawaharlal Nehru University, DELHI) to determine the strength of interaction between PKM2 and Histone H3. The sensor surface was eventually activated by N-hydroxysuccinimide (NHS, 0.05 M)/N-ethyl-N-(diethylaminopropyl) and carbodimide (EDC; 0.2 M). PKM2 diluted in sodium acetate buffer at pH 5.0 at a concentration of 100 nM was immobilized to the activated sensor surface. Two channels one as test and the other allegedly as control were employed for the experiment. PKM2 was immobilized in both channels, however Histone H3 was passed only through the test channel while buffer was passed through the second control channel. Post ligand immobilization, 100 mM ethanolamine at pH 7.4 was used for blocking and later 50 mM NaOH was used for the regeneration. Running buffer composition was as following: 10 mM HEPES [pH 7.4], 150 mM NaCl, 3 mM EDTA, and 0.05% NP-40 surfactant. The association kinetics for PKM2 was monitored for 400 s, followed by dissociation kinetics for 300 s. Different concentrations of Histone H3 (100 nM- 1000 nM) diluted in running buffer were injected across the sensor surface. SPR signals and resultant sensogram for PKM2-Histone H3 interactions were analyzed. Resultant sensogram was analyzed by subtracting change in the signal from activated/blocked control panel from that obtained as a consequence of binding of histone H3 to PKM2. At last the surface was regenerated with 75 mM NaOH. All the data were recorded at room temperature (i.e., 25°C). Autolab SPR Kinetic Evaluation software was used for data analysis.

### Exonuclease III assay

#### Nucleosome binding

500 nM of PKM2 was incubated with 100 nM of FAM-labeled nucleosome on ice overnight. Buffer composition was as 20mM HEPES buffer, pH 7.6, 50mM KCl, 5mM MgCl_2_, 5% sucrose, 0.1mg/ml of BSA, and 1 mM DTT.

Varying concentration of ExoIII enzyme was added to the 10ul of reaction mixture. After incubation at 23 °C reaction was terminated with 2X stop buffer containing 20mM EDTA and 2% SDS and addition of ~20μg of glycogen. DNA was extracted using phenol–chloroform–isoamyl alcohol (25:24:1). The samples were subjected to denaturing electrophoresis in a 7% polyacrylamide—7 M urea gel in 1X TBE buffer. The gel was scanned by using Typhoon Phosphorimager FLA9000 (GE Healthcare).

### Nucleosome sliding assay

Nucleosome sliding experiment was performed using an electrophoretic mobility shift assay (EMSA)-based method as described previously [45–47] with few modifications in the protocol. Buffer composition for nucleosome sliding was as following: 20mM HEPES buffer, pH 7.6, 50mM KCl, 5mM MgCl2, 5% sucrose, 0.1mg/ml of BSA, and 1 mM DTT. Nucleosome and remodeler were incubated at 23°C for 15 min and the sliding activity was initiated by adding 2.5mM ATP to the reaction mix. Reactions were quenched at the different time points by the addition of stop DNA (up to 25 μg of plasmid pJ201 containing a 34-mer array of 601 DNA sequences) in a buffer having composition as 25 mM EDTA in 20 mM HEPES pH 7.5, 50 mM KCl, 0.1 mg/ml of BSA, 5% sucrose, and 1 mM DTT and immediately placed on ice to halt the activity. The samples were then loaded onto a pre run 7% native polyacrylamide gels (60:1 acrylamide: bis-acrylamide) and electrophoresis was carried 120 V for 3 hr at 4°C. Gels were visualized using a Typhoon FLA9000 imager (GE Healthcare) and analyzed with ImageJ software (National Institutes of Health, Bethesda, Maryland, USA).

### Restriction enzyme digestion

To study the effect of PKM2 on nucleosome accessibility at chromatin level restriction digestion of pre-assembled nucleosome array of plasmid was carried out using HhaI enzyme. HhaI digestion site **GCG⌃C** has been inserted at dyad position 73 after each 208 bp and is usually obscured in nucleosome. Remodeling results in exposed restriction site and subsequent digestion of the DNA. To summarize 5nM of the pJ201 (34 × 601) chromatinized plasmid was incubated with 50 nM of Chd7 remodeler and 2.5mM of ATP with or without PKM2 at 23°C for 120 mins. Buffer used was 20mM HEPES, pH 7.6, 50mM KCl, 5 mM MgCl2, 1 mM EDTA, 1mM DTT, 0.1 mg/ml of BSA. Post incubation restriction digestion was performed using 1 U of HhaI enzyme for a time period of 30 min. The reaction was allowed for the defined time and stopped using Glycogen stop buffer (20 mM EDTA, 0.2 M NaCl, 1% sodium dodecyl sulphate and 0.25 mg/ml of glycogen). Samples were loaded on to 1.4% agarose gels. Electrophoresis was carried at 120V for 2hrs and gel was stained with ethidium bromide [48].

## Results

### Pyruvate Kinase M2 intrinsically binds Histone H3 to form a complex with the nucleosome core particle

**Fig 1:**
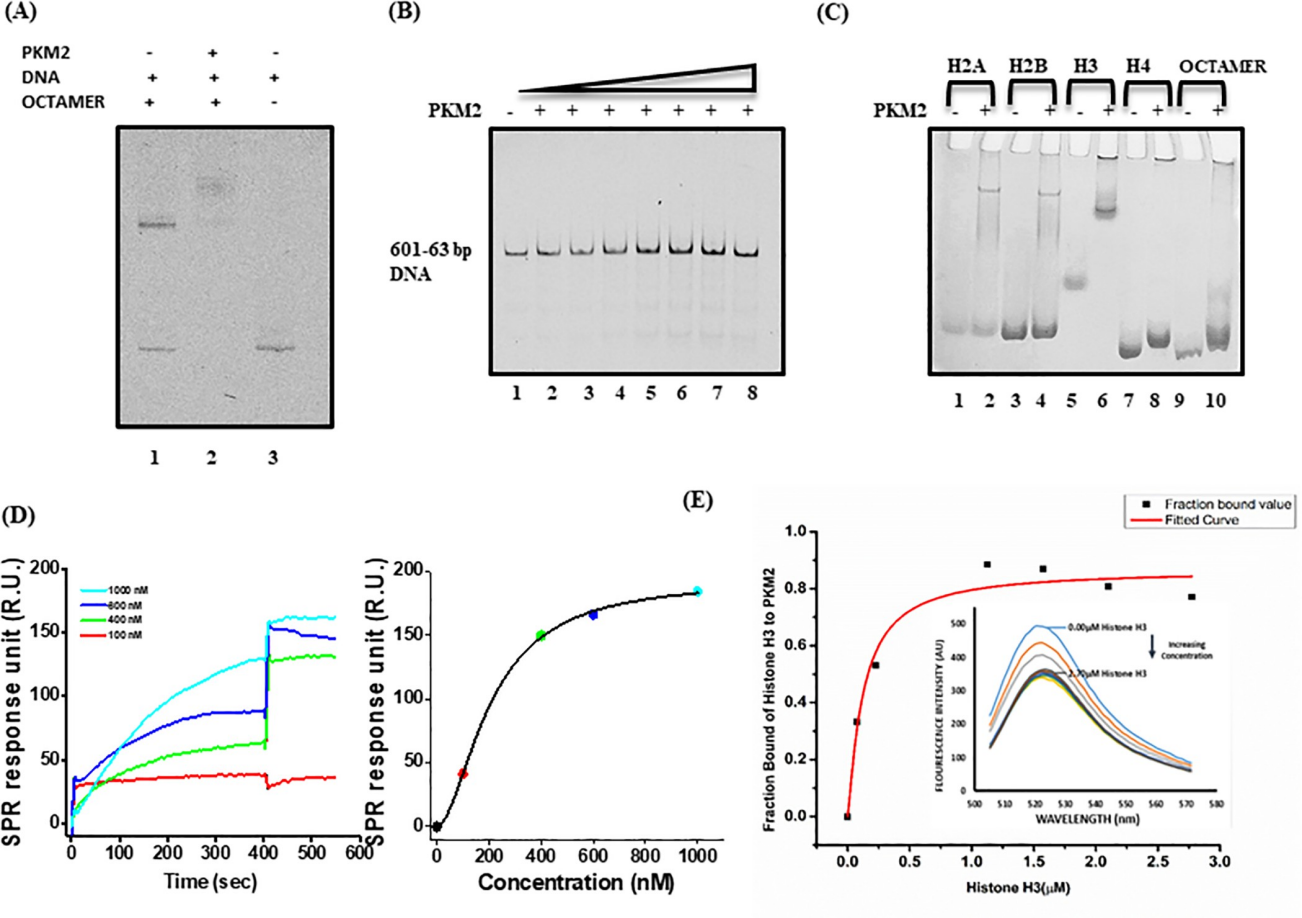
PKM2-nucleosome interaction. (A) Native PAGE electrophoresis of reconstituted complexes. The Nucleosome assembly is not independent of PKM2. (Lane1) Reconstituted FAM labelled mononucleosome after gradient salt dialysis (2M KCl-200mM KCl). DNA: octamer ratio 1:1.5.DNA working concentration is 5μM. (Lane 2) PKM2 bound to nucleosome complex with probable interaction through Histone H3. In reconstitution the Octamer: PKM2 ratio is 1:1.2. The reduced mobility on the native PAGE suggest a high molecular weight complex. No mononucleosme band observed. (Lane3) Free 208bp 5’-FAM labelled DNA. (B) Native Page electrophoresis of overnight incubated samples of 50 nM of fluorescent labelled DNA with different titration of PKM2 (1:1–1:10). There is no DNA-PKM2 interaction as can be inferred from no shift in the DNA bands on the native PAGE. (C) Interaction of PKM2 with Histone H3 was confirmed from binding assay. 50 μM of PKM2 was incubated with recombinant Histones (H1, H2, H3, H4 and histone octamer) in equimolar ratio to identify the potential interacting partner. Samples with and without PKM2 were loaded onto the gel and the bands were stained with coomassie after the electrophoresis. PKM2 interacted with Histone H3 as was evident from the shift in the band. (D) SPR sensogram plot fitted using 1:1 Langmuir model showing association and dissociation phase kinetics. Each concentration of the Histone H3 (analyte) is represented using different color for the corresponding binding curves. In this study, PKM2 (ligand) was immobilized on a sensor chip and different concentrations of Histone H3 (analyte) in running buffer was passed over ligand for kinetic evaluation. Increase in the sensogram response with increasing analyte concentration is observed from the graph plot as shown in the model. Fluorescence spectroscopy experiment using FITC-labelled PKM2 show probable interaction with the Histone H3 (ligand). (E) A gradual change in fluorescence intensity is observed with corresponding increase in the Histone H3 concentration. No change in the intensity is noted at higher concentration of ligand as seen from the plotted graph. This can be attributed to the quenching of fluorescence as a consequence of ligand binding. A saturation is reached when all the binding sites of PKM2 are occupied and no change in fluorescence intensity is obtained.

As previously reported oncogenic stimuli triggers tetramer-dimer switch which culminates into nuclear translocation of PKM2. Here we attempt to understand the implications of PKM2 through in vitro nucleosome reconstitution. Sequential binding of one unit of (H3–H4)2 tetramer and two units H2A–H2B dimers onto the Widom 601 nucleosome positioning sequence DNA is critical for the nucleosome assembly. Microscale reconstitution by employing Salt gradient dialysis with 1:1.5 DNA-to-octamer ratio as previously determined and subsequent titrations by varying the molar ratio PKM2 envince that nucleosome reconstitution can be possibly altered by PKM2 interaction. As can be inferred from native PAGE electrophoresis at a 1:2.4 PKM2-to-octamer ratio PKM2 interacts with the nucleosome and forms a complex of higher molecular weight (Fig 1A, Lane 2) compared to the suggested ensemble of histone octamer and the 601-0-63bp DNA (Fig 1A, Lane 1). To further define the nature of interaction we performed binding assay for PKM2 and the PrepCell (Bio-Rad) purified Widom 601 DNA. 50 nM of the 5’- FAM labelled DNA was incubated overnight with varied concentration (1:1 to 1:10 fold) of PKM2 on ice. However, no valid interaction was observed between the Widom 601 DNA and PKM2 (Fig 1B). We next examined the interaction of PKM2 with the different recombinant histones. All the four different Histones H2A, H2B, H3 and H4 were incubated overnight with PKM2 in 1:1 molar ratio. Results from the native gel electrophoresis validate a potential interaction between Histone H3 and PKM2 (Fig 1C). There is some observable interaction with histone H4 also but the interaction with histone H3 is more pronounced. This is in accordance with the previously reported pull down study between the two proteins [34]. We performed SPR with a range of concentrations (100nM-1000nM) of analyte Histone H3 for the kinetic evaluation of PKM2-Histone H3 binding. SPR is usually performed to determine the kinetic constants of label-free biomolecular interaction. It is important that the sensogram should be fitted to a kinetic model using a mathematical algorithm [49]. For our experiment the sensogram plots (Fig 1D) showing the association and dissociation phase was derived using Langmuir curve-fitting model which describes 1:1 interaction of ligand and the analyte. There is subsequent increase in the response unit associated with the interaction of Histone H3 to the immobilized PKM2 and the interaction apparently seem very stable. However, measure of *k*_d_ value of very stable interactions is difficult and at the same time might be misleading under mild or physiological conditions. The complex decay in such cases is extremely slow and *k*_d_ value should be only a qualitative measure and not quantitative. We can conclude from the aforementioned binding parameters that immobilized PKM2 interacts with Histone H3 and there is a gradual increase in the response unit with increase in Histone H3 concentration. A kinetic equilibrium is obtained represented by plateau in the association phase of the plot. Affinity Constant (KD) value for the binding kinetics is 6.39E-06M (Affinity error 3.20E-07) and calculated from the equilibrium equation. The obtained value is mean of the experiment repeated in triplicate. However the rate of complex decay is remarkably low as we do not observe any significant dissociation of the PKM2-Histone H3 complex suggesting that some other factor might be needed to break the association between the two biomolecules. This dissociation phase kinetics is interesting and needs to be further investigated. Fluorescence spectroscopy experiment using FITC labelled PKM2 and Histone H3 further validate the interaction between the two proteins. Interestingly we observe a decrease in the fluorescence intensity of labelled PKM2 when titrated with increasing concentration of Histone H3. With subsequent increase in the Histone H3 concentration in the PKM2 microenvironment the fluorescence is gradually quenched and a steady depreciation in the FITC fluorescence intensity is recorded which finally becomes stabilized and no further change in the intensity corresponding to higher concentrations of Histone H3 is observed (Fig 1E). The Dissociation constant (KD) as obtained is 8.03 ± 0.03M (Mean of the experiment done in triplicate).We conclude that Histone H3 probably quenches the fluorescence by binding to PKM2 and with increasing concentration binding is further enhanced thus exerting a negative effect on the fluorescence intensity. However, as the binding sites of PKM2 get sequentially occupied by PKM2 an equilibrium is reached wherein no more interaction is possible. This causes a saturation with no notable change in the fluorescence spectra.

### Exonuclease III digestion of pyruvate kinase M2- NCP complex

**Fig 2:**
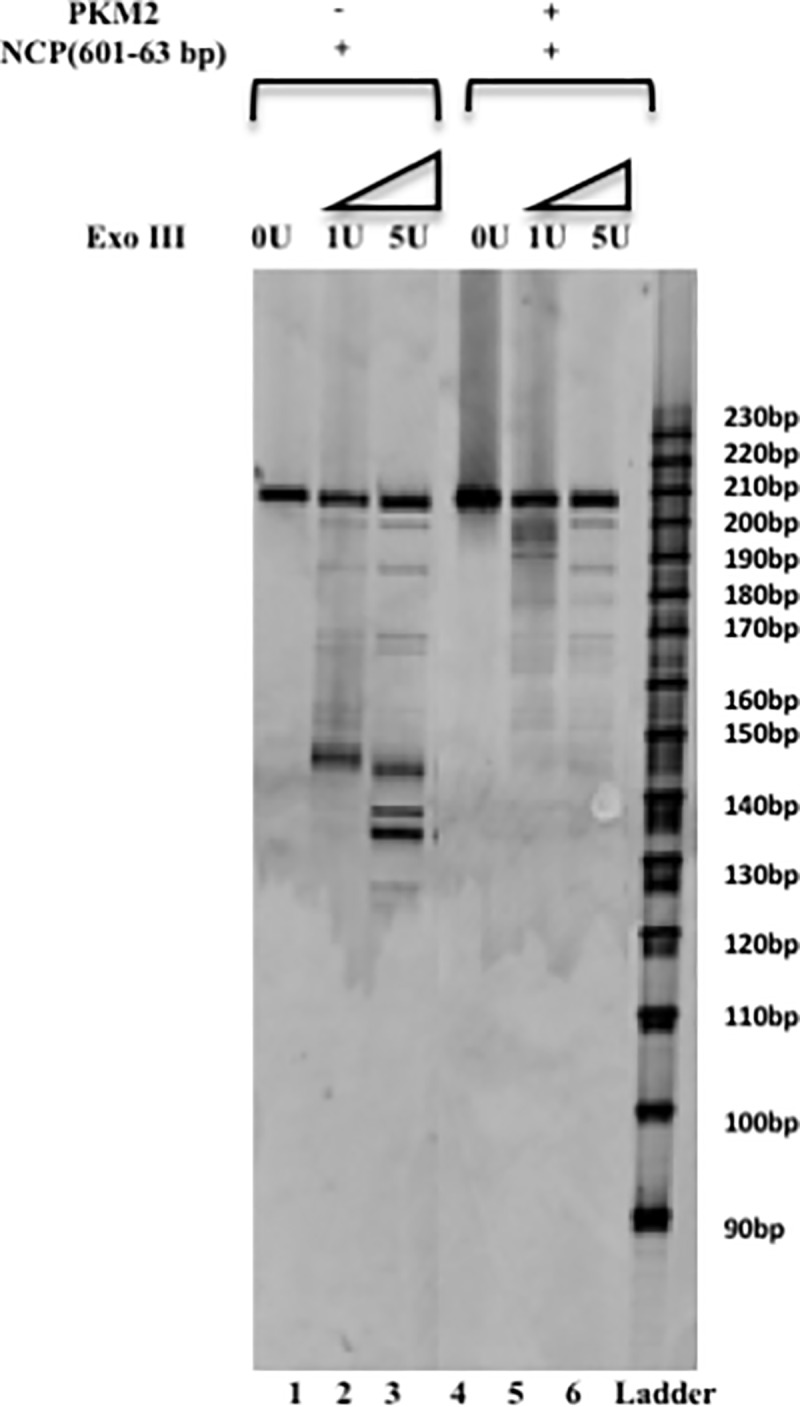
DNA accessibility test with Exonuclease III. PKM2 interacts with nucleosome and reduces the accessibility. To prove this 100 nM of FAM labelled nucleosome was incubated with 500 nM of PKM2 in the presence of binding buffer. The sample were treated with varying concentration of ExoIII enzyme which yields DNA fragment that vary by 10 bases. Constrained digestion with 1U of ExoIII yields a band of 145 bp core and at higher concentration of ExoIII the DNA bases of the core complex is also chopped yielding smaller fragments. PKM2-nucleosome complex hinders the ExoIII activity and the digestion of the nucleosome is obstructed. Significant reduction in the chopped DNA bands is observed suggesting that PKM2 reduces access to nucleosome.

Exonuclease III possess the phosphomonoesterase activity and catalyzes the 3' exonucleolytic digestion of the duplex DNA. The activity is hindered by the DNA-histone interaction and at regulated low levels of ExoIII, digestion of the nucleosome monomers yield a compact core of 145 bp which defines the boundary of the nucleosome. On extensive ExoIII digestion the nucleosome core is further chopped to yield discrete bands that are integer multiples of 10 bases signifying sequential disruption of the DNA–histone contacts at every turn of the helix with the advance of ExoIII [49]. We used varied concentration of ExoIII (1U and 5U) for our experiment. It was observed that nucleosome and nucleosome-PKM2 complex yield a 208 bp DNA band in the absence of any restriction digestion. 1U of ExoIII exhibits a controlled digestion of complex and yield a 145 bp of DNA reflecting the digestion of the 63bp linker DNA that does not form the Nucleosome core (Fig 2 left side). Increasing the concentration of the ExoIII leads to a more extensive digestion pattern and results in several digested bands varying in 10 bases. For 5U of ExoIII enzyme the bands correspond to approximately 120 bp of DNA, suggesting the nucleosome is breached and the enzyme is able to cleave beyond the core boundary. For PKM2-nucleosme complex the digestion of the nucleosome is remarkably impaired as can be inferred from the digestion profile (Fig 2 right side). Interestingly there is no significant effect of higher concentration of enzyme as we do not observe any augmented digestion of the nucleosomal DNA. This is intriguing since ExoIII chops double helix DNA and activity is suspended only by the histone-DNA interaction. As shown before (Fig 1B) PKM2 does not interact with DNA. However, our result with restriction digestion contradict this finding. To conclude we can infer that PKM2 when complexed with the nucleosome obscure the core and hinder the passage of ExoIII. Henceforth, we propose that PKM2 interacts with nucleosome and thereby reduces the ExoIII enzyme access to the complex.

### Pyruvate Kinase M2 can potentially disrupt the ChD7 mediated remodeling of nucleosome

**Fig 3:**
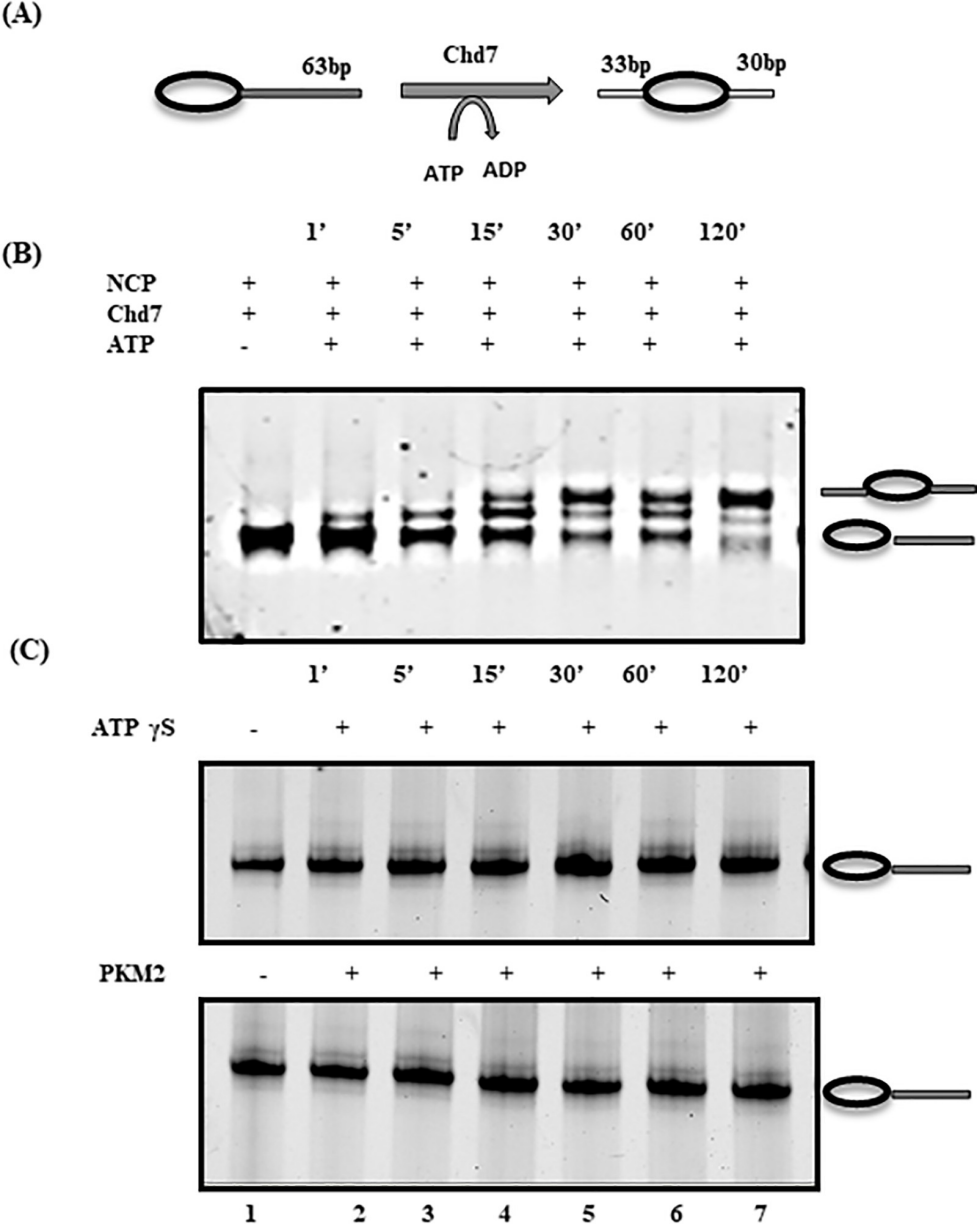
PKM2 blocks ΔChd7 mediated nucleosome sliding. (A) Schematic representation of the ATP dependent nucleosome remodeling activity of ΔChd7. The Chd7 remodeler has DNA-binding domains namely SANT and SLIDE and an ATP motor domain. ATP hydrolysis catalyzes the alteration in the nucleosome structure by the sliding the end positioned octamer towards the center. (B) Sliding Assay for establishing the remodeling activity of ΔChd7. 50 nM of end labelled mononucleosme was incubated with 10 nM of ΔChd7. Catalytic activity was triggered by addition of 2.5mM of ATP. Reaction was quenched at varying time point (1’, 5’, 15’, 30’, 60’, and 120’) and the samples were loaded onto native PAGE for electrophoresis. It can be noted that after 120 mins of incubation majority of the nucleosome were positioned at the center. (C) ΔChd7 mediated remodeling activity is completely impaired in the presence of “nonhydrolyzable” ATP analog ATPγS. No sliding of the end positioned nucleosome is observed at any of the predefined time points. Similar results were obtained when sliding assay was performed in the presence of PKM2 (500nM) suggesting that PKM2 inhibits nucleosome remodeling.

As evident from the restriction digestion assay PKM2 interacts with nucleosome and reduces the accessibility to the complex. We were interested in exploring the possible outcomes of this finding and how it might contribute to altered gene expression. Chd7 is a member of the SNF2-protein superfamily and is reported to play a role in DDR (DNA Damage Repair). To cite as per the reports any aberrant expression or somatic mutations of Chd7 often in many cases may lead to cancer. Chd7 exhibits an intrinsic ATP-dependent nucleosome remodeling activity by sliding the histone octamer from one end of the DNA fragment to the centered position (Fig 3A). This subtle alteration in the position is reflected by varied mobility on a native PAGE electrophoresis. We performed the nucleosome Sliding Assay with ΔChd7 and observed dynamic shift in the nucleosome position at various time point of 1min, 15min, 30min, 60min and 120 min. It was observed that with time the nucleosome conformation was dynamically altered and post 120 min a significant population of nucleosome octamer was positioned from the end of the DNA fragment towards the center (Fig 3B, lane7). The propensity of nucleosome sliding is completely depreciated when ATP was replaced with “nonhydrolyzable” ATP analog ATPγS (Fig 3C). To study the possible effect of PKM2 on the sliding activity of ΔChd7 we carried out the Sliding experiment in the presence of PKM2. Sliding was allowed and later quenched after the defined time duration as previously monitored. It was interesting to note PKM2 completely obstructed the nucleosome sliding and we could not detect any shift in the nucleosome position. From our native PAGE binding experiments (S1 Fig) it was observed and deduced that PKM2 failed to interact with ΔChd7. Thus, the abrogated ΔChd7 activity can forthrightly be attributed to the interaction of PKM2 with the nucleosome.

### Interaction of PKM2 with the 34-mer 601 array

**Fig 4:**
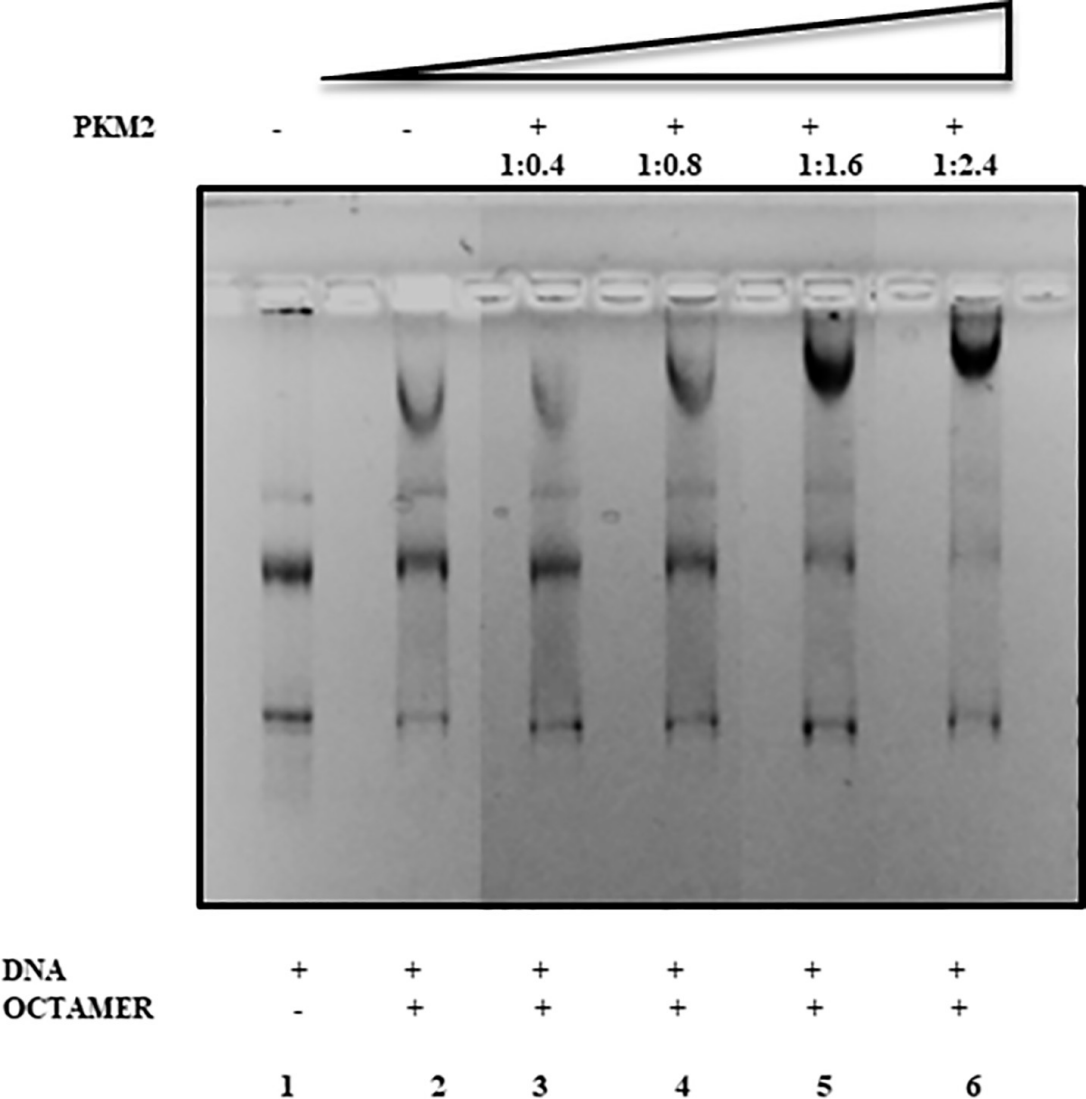
PKM2 interacts with chromatin. Chromatin reconstitution using pJ201 (34 × 601) plasmid construct in the presence of PKM2 yield chromatin-PKM2 complex. Chromatin reconstitution was achieved by gradient salt dialysis. Result with different working ratio of octamer-PKM2 suggest that PKM2 actively interacts with chromatin. Interaction is observed at 1: 0.4 ratio which gradually increases and is noted to be maximum at 1:2.4 ratio.

Since DNA exist as chromatin in the nucleus and DNA-directed processes have majorly evolved with the chromatin [50] we further extended our nucleosome binding results to the level of chromatin organization. For the chromatin reconstitution we employed the described protocol of Routh and Rhodes. pJ201 (34 × 601) [48] with 208 bp-tandem repeat plasmid was incubated with octamer (1μM) in the presence of different titrations of PKM2 in high salt buffer during the chromatin reconstitution stage. The mixture was subjected to gradient salt dialysis [37]. Agarose gel electrophoresis of the complex mixture in accordance with the nucleosome binding experiments show the sequential formation of PKM2- chromatin complex. We observe binding at the 1:0.4 Octamer to PKM2 binding ratio (Fig 4, lane 3) which gradually saturates with maximum binding at 1: 2.4 (Fig 4, lane 6), thus it can be inferred that PKM2 possibly interacts with the chromatin to form a complex. It may be deduced that PKM2 is getting incorporated in chromatin in some fashion which needs to be investigated to gain a deeper mechanistic insight.

### PKM2 impairs Chd7 remodeling in a chromatin array and negatively impact nucleosome positioning

**Fig 5:**
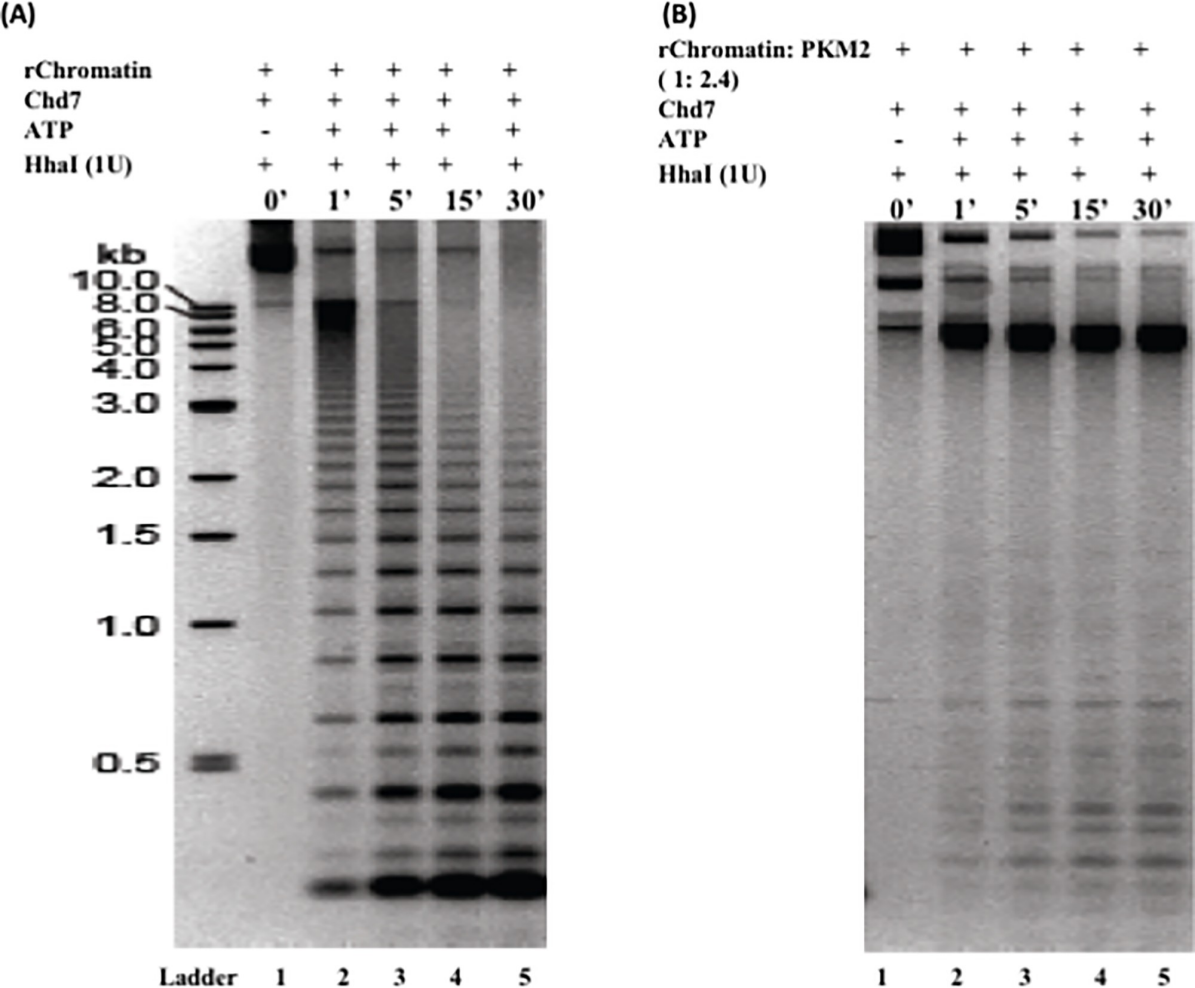
PKM2 negatively modulate chromatin remodeling. ΔChd7 causes nucleosome repositioning in a chromatin array resulting in exposed HhaI sites at the dyad position. HhaI sites are obstructed by the nucleosome. When chromatinized plasmid is incubated with 50 nM of ΔChd7 remodeler and 2.5mM of ATP it results in nucleosome repositioning exposing the HhaI sites. The remodeled chromatin is digested by HhaI (IU) to yield DNA fragments and the enzyme activity is positively correlated with the incubation time. Maximum digestion is obtained post 30 min of incubation. As in the case of mononucleosome chromatinized plasmid incubated with PKM2 do not exhibit remodeling and HhaI digestion is impaired due to the absence of exposed restriction sites.

To dissect the impact of PKM2 on nucleosome positioning in a chromatin array and investigate if it could hinder Chd7 driven remodeling we used 34-mer nucleosome array for our study. The pJ201 (34 × 601) construct has restriction site for HhaI at the dyad position. The 34-mer reconstituted chromatin when digested by HhaI do not show any digested fingerprint (Fig 5B, lane1) suggesting the site is obstructed by DNA-octamer interaction. ΔChd7 mediated nucleosome sliding of the chromatin results in exposed HhaI sites which are subsequently digested to yield bands similar to that of naked DNA. The proclivity for HhaI digestion notably increases with time and evident difference can be observed in the digestion fingerprint with varying time of incubation. It can be surmised (Fig 5A, lane 5–6) that significant fraction of the HhaI sites are cleaved with majority release of 208bp DNA fragment after 30 min of incubation at 23°C. To assess the impact of PKM2 on the chromatin structure we performed HhaI digestion of the reconstituted chromatin with PKM2 for 30 min at 23°C. In concurrence with our mononucleosme result the chromatinized plasmid could not undergo chromatin remodeling when incubated with PKM2 as can be observed from the lack of digested bands. To conclude ΔChd7 causes nucleosome repositioning and as a consequence the HhaI site is exposed which can be cleaved by the enzyme action. However, PKM2 when interacts with chromatin hinder the activity of ΔChd7. Subsequently the remodeler fails to slide the octamer, the sites for digestion are obscured and inaccessible to the restriction enzyme and thus digestion of the chromatin is inhibited. This is significant observation and implicate PKM2 as an opposing factor to nucleosome remodeling.

## Discussion

Here, we report that PKM2 potentially acts as a modulator of chromatin remodeling activity by directly interacting with chromatin and thus contribute to oncogenesis. We highlight a rather unaddressed role of PKM2 that may be pivotal in exacerbating genomic integrity and trigger cell proliferation. The metabolic functions of PKM2 as key catalytic enzyme of glycolytic cycle and regulator of the energy flux in a cell is well established. However, the non-metabolic functions of PKM2 and its various aspects need to be discerned. The oligomeric switch as a consequence of oncogenic signal mediated post-translational modification subsequently facilitate nuclear translocation. Using fluorescence labelled NCP (nucleosome core particle) and 34-mer chromatin array we have demonstrated direct interaction of PKM2 with the nucleosome complex. We examined the possible interaction of PKM2 through nucleosome reconstitution. Gel mobility shift assay result of the reconstituted complex validate the potential binding of PKM2 to nucleosome. It can be inferred that PKM2 may have a role to play in nucleosome assembly and thereby dictate a likely control over gene expression. However the implications of this interaction is immense and needs to be further explored. Intuitively the nature of this interaction was further verified by establishing an evident association of PKM2 with Histone H3. Employing native PAGE gel electrophoresis we confirmed that PKM2 binds with Histone H3 and there is a possible interaction with Histone H4 (as seen in the native PAGE) which was although not as promising as Histone H3. The possibility of interaction with Histone H4 is a subject of study and further interpretation.

Our finding was consistent as confirmed by SPR and fluorescence spectrometry experiments. This study was further extended to the validation of 34-mer chromatin array emphasizing the veracity of this interaction to the chromatin level. The results further prove the possibility of wider involvement of PKM2 in the chromatin structural organization and the downstream implication it may exert on gene regulation.

Nucleosome restrict the accessibility of transcription factors and impede transcription and other chromatin transactions. This can be attributed to the 14 contact points between DNA and the histone octamer. However, protein complexes like chromatin remodelers can dynamically reposition and slide nucleosome to alter gene expression suggesting multi-layered regulation [21]

To dissect the impact of PKM2 on nucleosome accessibility we performed Exonuclease III digestion of PKM2-Nucleosome complex. Constrained digestion of nucleosome at low concentration of enzyme yield a nucleosome core with 145 bp wrapped around it which is further cleaved with the increase in enzyme units. However, in case of PKM2 bound nucleosome it is remarkable to observe from the lack of cleaved bands on the denaturing gel that digestion cannot proceed and the enzyme activity is significantly compromised. Results suggest that PKM2 probably impose a barrier to the remodeling enzymes, insulate nucleosome and obstruct access.

As previously noted, in presence of nucleosome, transcription machinery face a roadblock and the highly coordinated process of gene regulation is impaired. Several chromatin remodelers relieve this transcriptional blockade by facilitating nucleosome sliding. CHD family of remodelers harness the energy generated from ATP hydrolysis to alter the chromatin structure. The interplay between the repair machinery and the remodeler is crucial to gain an access to DNA double-strand breaks (DSB) and prevent genomic instability [51]. Chd7 is an ATP dependent chromatin remodeler and implicated in transcriptional regulation, DNA repair etc. It induces alteration in the Histone-DNA contact and repositions end labelled nucleosome towards center. This ATP dependent activity of Chd7 which is impaired when ATP is replaced with “nonhydrolyzable” ATP analog ATPγS was shown in our electrophoretic mobility assay. It is well acknowledged fact that altered metabolism catapults cell towards a more proliferative state and PKM2 plays a central role in this transition. Here, we have addressed how PKM2 a metabolic enzyme can exert control on the gene expression by modulating the nucleosome positioning. It was observed that ΔChd7 failed to slide the end positioned nucleosome towards the center in the presence of PKM2. The remodeling activity of ΔChd7 was completely thwarted suggesting that there is negative co-relation between PKM2 and ΔChd7 nucleosome sliding activity.

Nucleosome mapping reactions with HhaI using 34-mer pre-assembled nucleosome array imply that PKM2 impair remodeling in chromatin without altering the structure. In contrast to chromatin digestion in the presence of the ΔChd7 which was on account of the exposed HhaI site at the dyad position we fail to observe any digestion in presence of PKM2. This was evidently on account of inhibition of nucleosome sliding.

**Fig 6:**
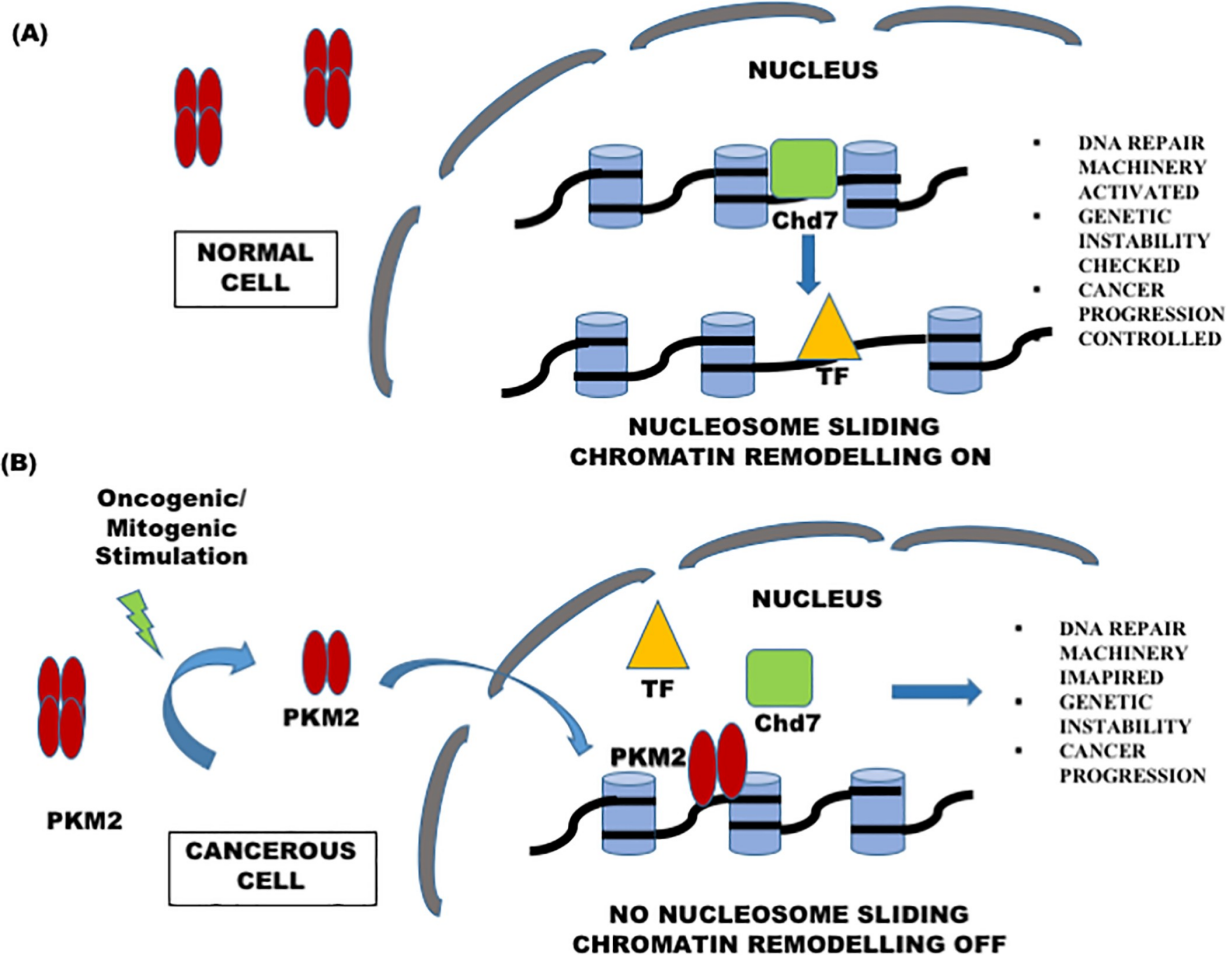
Proposed mechanism based on the study. Nuclear PKM2 can directly alter the expression of genes by hindering nucleosome positioning and inhibiting remodeling. PKM2 obscure nucleosome and create a roadblock for the Chd7 remodeler and thus impede nucleosome sliding. Chd7 is implicated in a number of developmental disorder and is reported to play a role in cancer as well. As an important factor for DNA Damage Repair machinery any impairment in its functions can have detrimental implications. We hypothesise that PKM2 by negatively modulating Chd7 catalysed chromatin remodeling can exacerbate genomic instability and in turn contribute to cancer.

To summarize, we have shown that PKM2 can directly interact with the nucleosome through Histone H3 and obscure the chromatin from undergoing epigenetic modifications catalyzed by remodeling complexes. The basis of this prediction was evident from the suspended remodeling activity of Chd7 in the presence of PKM2 which can possibly lead to dysfunctional DDR machinery and genomic instability (Fig 6). As per this study association does appear to have impaling consequence on the nucleosome remodeling and probably on the subsequent transcriptional process which need to explored and is a subject of further investigation. It is noteworthy that this aspect of layered gene regulation is unique to the understanding of cancer and further implicate PKM2 and its impact on tumorigenesis.

## Conclusion

In a cancer cell metabolic overhaul often commensurate with dysfunctional metabolic enzymes that synergistically impact the epigenetic blueprint and promote altered gene expression. In this research article we report a so far unaddressed role of PKM2 as a modulator of chromatin remodeling activity. Our study show that PKM2 can directly interact with nucleosome through Histone H3 and inhibit Chd7 mediated remodeling. At present non-metabolic functions of PKM2 is an intensely researched domain and its role in cancer progression and tumorigenesis is widely being acknowledged. Besides acting as a key nuclear kinase it is known to regulate the expression of several oncogenes. Here, we attempt to answer how PKM2 can directly alter the expression of genes by hindering nucleosome positioning and inhibiting remodeling. Exonuclease III digestion of Pyruvate Kinase M2- NCP complex suggest that PKM2 obscure nucleosome and create a roadblock for the Chd7 remodeler and thereby impede nucleosome sliding. Chd7 is implicated in a number of developmental disorder and is reported to play a role in cancer as well. As an important factor for DNA Damage Repair machinery any impairment in its functions can have detrimental implications. It can be inferred that PKM2 by negatively modulating Chd7 catalyzed chromatin remodeling may probably exacerbate genomic instability and in turn contribute to cancer. Through this study we aim to highlight the impact of PKM2 on chromatin architecture and its impact on the epigenetic landscape of the cell.

## Supporting information

S1 Fig Gel mobility shift assay. (A) Interaction of Chd7 with PKM2 with increasing molar concentration of PKM2. (B) Interaction of PKM2 with Chd7 with increasing molar concentration of Chd7.
(PDF)
