# Supplementary material for: Pyruvate Kinase M2 serves as blockade for nucleosome repositioning and abrogates Chd7 remodeling activity
Source: PLoS One. 2019 Feb 8;14(2):e0211515. doi: 10.1371/journal.pone.0211515 (PMC6368300; doi:10.1371/journal.pone.0211515)
Supplement: S1 Fig — (A) Interaction of Chd7 with PKM2 with increasing molar concentration of PKM2. (B) Interaction of PKM2 with Chd7 with increasing molar concentration of Chd7. (PDF) [file pone.0211515.s001.pdf]

SUPPLEMENTARY DATA

1. Gel mobility shift assay was performed to assess the interaction of PKM2 with Chd7. 1μM of Chd7 was incubated overnight at 4^0C on ice and titrated with increasing concentration of PKM2 (1:1, 1:5). Same set of reaction was employed for 1μM of PKM2 with molar ratio of Chd7 as 1:1 1nd 1:5. Buffer composition for nucleosome sliding was as following: 20mM HEPES buffer, pH 7.6, 50mM KCl, 5mM $MgCl_2$. 5 % Native PAGE gel was pre-run in chilled 0.25X TBE buffer for 30min at 90V .Samples were loaded onto the gel and the bands were stained with after the electrophoresis at 110V for 2hrs and subsequently stained coomassie dye. We do not observe any shift in the bands corresponding to either PKM2 or Chd7.

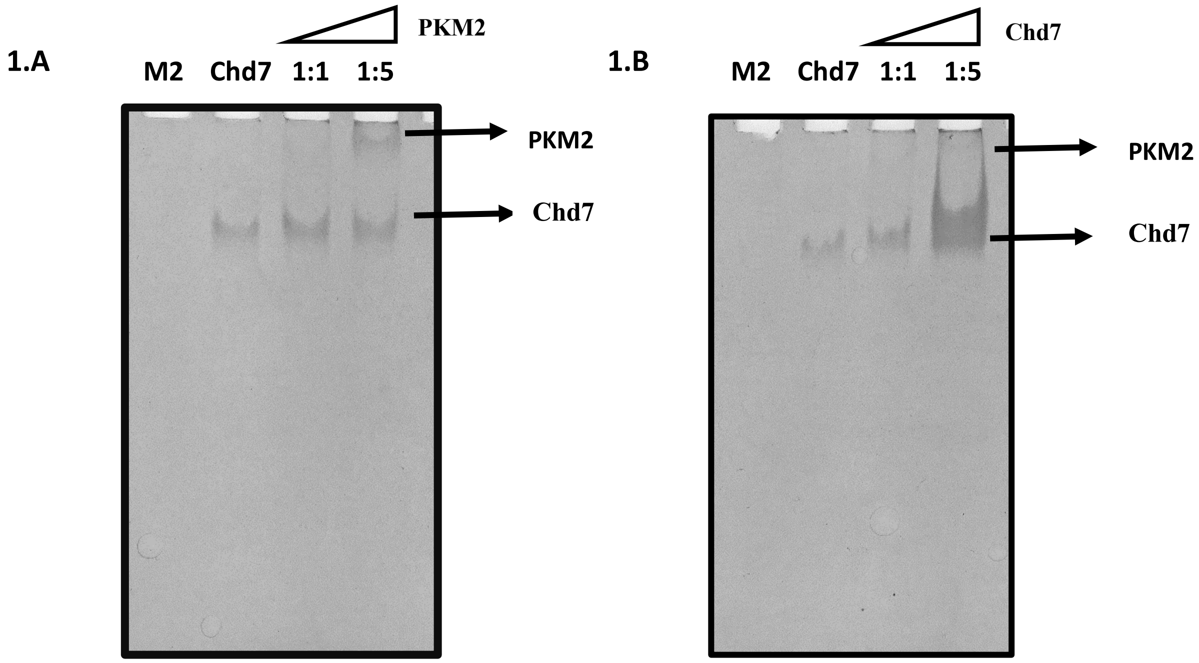

Fig 1. Gel mobility shift assay

(A) Interaction of Chd7 with PKM2 with increasing molar concentration of PKM2
(B) Interaction of PKM2 with Chd7 with increasing molar concentration of Chd7
